# Supplementary material for: Causal interplay between lactose intolerance and gut microbiota: a combined bidirectional Mendelian randomization and in vivo validation study
Source: Front Nutr. 2026 Jun 1;13:1803337. doi: 10.3389/fnut.2026.1803337 (PMC13265576; doi:10.3389/fnut.2026.1803337)
Supplement: Supplementary file 1 [file Data_Sheet_1.zip › supplementary materials/Forward/forest plot/ebi-a-GCST90027712.finngen_R12_E4_LACTONAS.pdf]

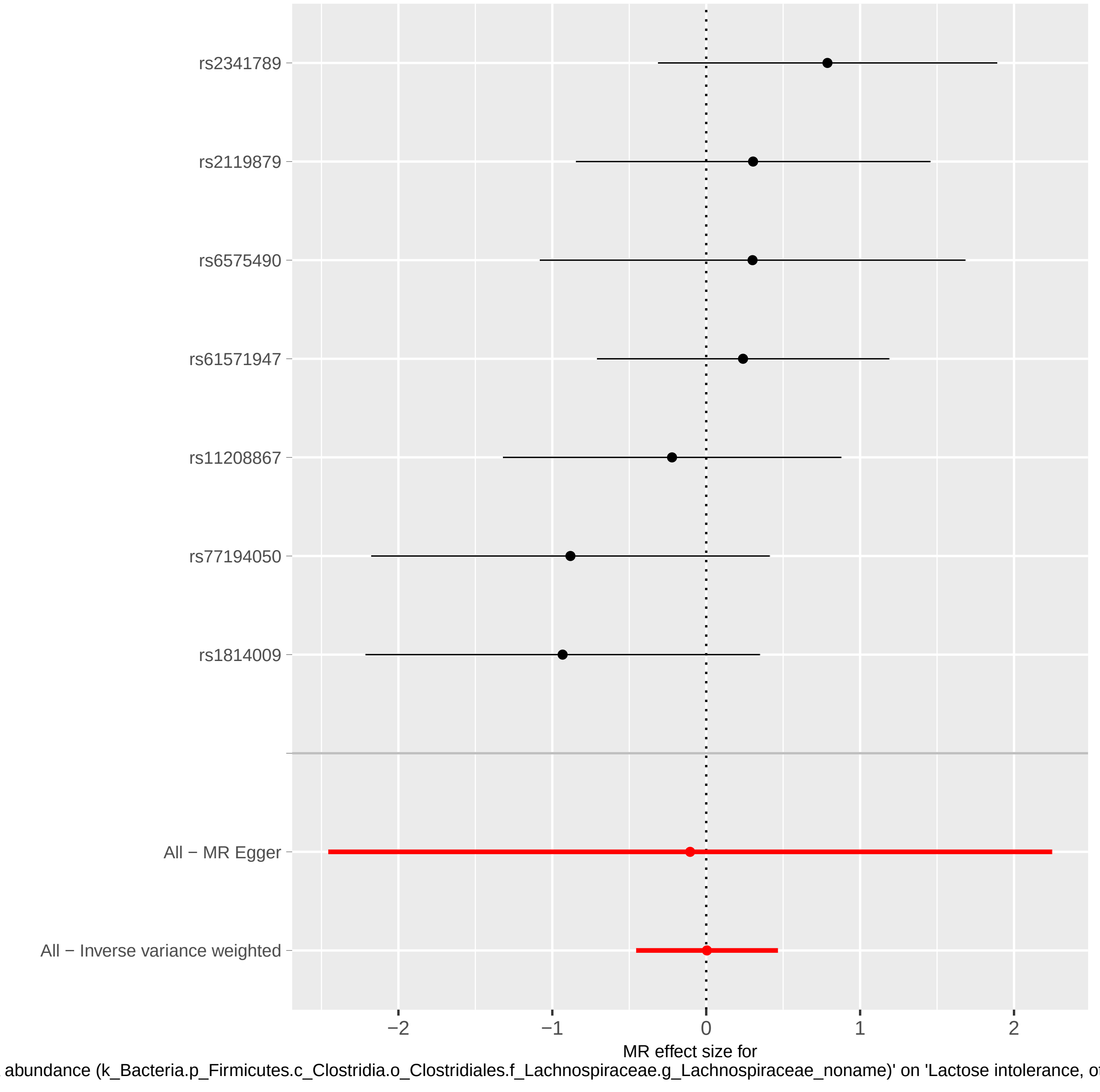
rs2341789
rs2119879
rs6575490
rs61571947
rs11208867
rs77194050
rs1814009
All − MR Egger
All − Inverse variance weighted
−2
−1
0
1
2
MR effect size for
abundance (k_Bacteria.p_Firmicutes.c_Clostridia.o_Clostridiales.f_Lachnospiraceae.g_Lachnospiraceae_noname)' on 'Lactose intolerance, o
